# An unusual endobronchial lesion: expanding the differential diagnosis

**DOI:** 10.1002/rcr2.429

**Published:** 2019-04-19

**Authors:** Rachel Leonard, Charles Schultz, Sarah Hadique

**Affiliations:** ^1^ Section of Pulmonary, Critical Care and Sleep Medicine, Department of Internal Medicine West Virginia University Morgantown West Virginia USA; ^2^ Department of Pathology West Virginia University Morgantown West Virginia USA

**Keywords:** Endobronchial lesion, lung, synovial sarcoma

## Abstract

Synovial sarcoma is a rare tumour, accounting for approximately 2.5–10% of all soft tissue sarcomas. In the thorax, it most often presents as a large, homogenous mass and, most commonly, is the result of extrathoracic tumour metastasis. Here, we report a case of a 73‐year‐old male who presented to the hospital after a motor vehicle collision. Chest computed tomography demonstrated a 2.0 × 2.4 cm left lower lobe pulmonary nodule with endobronchial extension and a 2.5 × 2.1 cm right‐sided kidney mass. He was eventually diagnosed with monophasic synovial sarcoma. To date, only seven other cases of primary pulmonary synovial sarcoma with endobronchial extension have been reported. A review of the cases and literature is discussed.

## Introduction

Synovial sarcoma is a rare soft tissue sarcoma. It most commonly occurs in young adults in the extremities, especially in the close proximity of large joints. Pulmonary sarcomas are exceptionally rare, and metastases from extrapulmonary sarcomas are undoubtedly more common than primary pulmonary sarcomas. A diagnosis of synovial sarcoma is dependent on histological, immunohistochemical, and chromosomal translocation testing. Here, we report a case of an incidentally found primary pulmonary synovial sarcoma with endobronchial extension in a 73‐year‐old man and provide a review of the literature.

## Case Report

**Figure 1:**
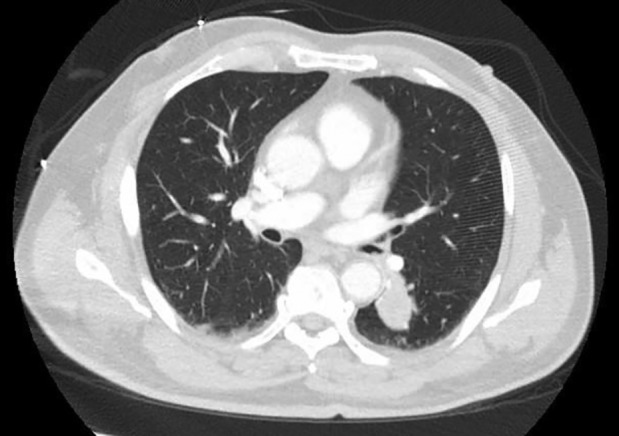
Axial computer tomography images of the chest demonstrate a left lower lobe nodule measuring 2.0 × 2.4 cm with endobronchial involvement.

A previously healthy 73‐year‐old man presented to the emergency department after a motor vehicle collision. Computed tomography (CT) of the chest, abdomen, and pelvis demonstrated a 2.0 × 2.4 cm left lower lobe pulmonary nodule with endobronchial extension and a 2.5 × 2.1 cm right‐sided kidney mass (Fig. 1). He was a life‐time non‐smoker and reported only occasional alcohol intake. He worked as a mechanic all his life. His only complaint was a cough, productive of yellow sputum without haemoptysis. He denied any fever, dyspnoea, weight loss, or night sweats. Positron emission tomography (PET) scan demonstrated a standardized uptake value (SUV) of 3.5 for the lung nodule and 5.1 for the renal mass.

**Figure 2:**
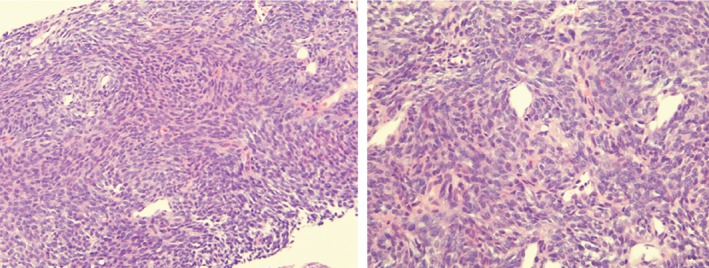
20× and 40× H&E‐stained sections of the initial biopsy. The tumour displays monotonous, densely cellular spindle cells in a vaguely fascicular pattern with a few small vessels.

**Figure 3:**
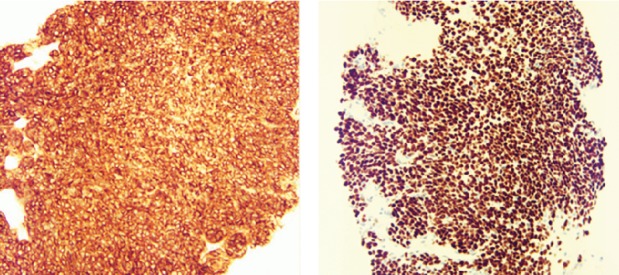
Tumour staining with strong positivity for BCL‐2 and TLE, respectively. This pattern is characteristic of synovial sarcoma.

Flexible bronchoscopy demonstrated a large endobronchial polypoidal mass lesion within the left mainstem bronchus. Forceps biopsies and fine‐needle aspirates were non‐diagnostic and demonstrated only necrotic tissue. The patient was advised to undergo a repeat bronchoscopy with cryobiopsies under general anaesthesia. However, he declined any additional sampling. He was also evaluated for thoracic surgery and refused resection of the lung mass. He presented 1 year later with worsening of cough and new‐onset dyspnoea. Repeat imaging demonstrated significant increase in his left‐sided pulmonary nodule (8.5 × 7.5 × 8.5 cm) with stable renal mass (2.0 × 2.0 cm). Our leading diagnosis was metastatic renal cell carcinoma (RCC) given the presence of a kidney mass on imaging. A CT‐guided biopsy of his left lower lobe lung mass was performed, and that demonstrated monotonous, densely cellular spindle cells in a vaguely fascicular pattern (Fig. 2). It stained strongly positive for BCL‐2, vimentin, and transducin‐like enhancer of split 1 (TLE1), which is characteristic of synovial sarcoma (Fig. 3). Polymerase chain reaction (PCR) demonstrated the presence of an SS18‐SSX1 fusion confirming the diagnosis. He underwent a video‐assisted thoracoscopic surgery (VATS) sleeve lobectomy with lymph node dissection, which was negative for metastatic disease.

Our patient did well post‐lobectomy. He subsequently underwent partial nephrectomy, with pathology demonstrating RCC. Follow‐up imaging five months after surgery showed no residual disease in the chest, abdomen, or pelvis.

## Discussion

Incidental findings on CT are common. In a series, 44.5% of CTs performed for trauma showed unrelated findings 1. A newly discovered pulmonary nodule or mass on chest CT in middle‐aged to elderly patients raises immediate concern for malignancy, and tissue biopsy is needed to identify underlying pathology. In our patient, several diagnostic possibilities were entertained, but primary lung cancer was an important consideration. Approximately 10–15% of lung cancers arise in life‐time non‐smokers, making it one of the leading causes of cancer‐related mortality in these patients 2. The presence of both a renal mass and lung nodule raised suspicion for RCC with solitary metastasis. Although it is typical to have multiple pulmonary metastases, a solitary metastasis is identified at the time of diagnosis of RCC in about 2–4% of patients 3.

Differential diagnosis also included other tumours such as primary tracheobronchial tumours and lymphoma and benign conditions such as amyloidosis, tuberculosis, and fungal infections that can involve airways and pulmonary parenchyma. Primary tracheobronchial tumours are rare, representing only 0.6% of all pulmonary tumours. Predominant primary malignant tracheobronchial tumours are squamous cell carcinoma, adenoid cystic carcinoma, and mucoepidermoid carcinoma 4.

Bronchoscopy is the usual approach to biopsy for pulmonary lesions with an endobronchial extension. However, bronchoscopy fails to provide diagnosis in 10–15% of endoscopically visible lesions, as seen in our case 5. Failure to identify the underlying pathology with bronchoscopy poses a diagnostic dilemma for clinicians. Further options include repeat bronchoscopy with additional biopsies, CT‐guided fine‐needle aspiration, or rigid bronchoscopy with core biopsies. There is an emerging role for cryobiopsies in such patients 5. It is critical to stress that the absence of malignancy on bronchoscopic biopsies cannot be accepted as evidence of benignity. Physicians must continue diligent efforts to identify true underlying pathology.

Synovial sarcoma is a rare tumour, accounting for approximately 2.5–10% of all soft tissue sarcomas 6. In early literature, it was believed to arise from synovium because it was frequently found around large joints, particularly the knee. Its name is a misnomer because the actual cells from which this tumour develops are unknown and are not necessarily synovial. Synovial sarcoma is associated with the presence of the t(X;18)(p11.2;q11.2) translocation, which is described in >95% of the cases 7. It occurs most commonly between ages 15 and 40 years, with peak incidence in the third decade. As much as 90% of cases present before the age of 60 years. Most tumours are located in the extremities as a solitary tumour and often cause localized pain and tenderness 8. Metastases occur in approximately 50% of cases. Usual metastatic sites are the lung, pleura, bone, and lymph nodes 6.

Primary pleuropulmonary synovial sarcoma (PPSS) only accounts for 0.1% of all lung tumours 9. Like other malignant mesenchymal tumours of the lung, synovial sarcomas usually metastasize from an extrathoracic tumour, and the exclusion of extrathoracic synovial sarcoma is essential to determine the pulmonary origin of the tumour 7. Typically, PSSS present either asymptomatically, as incidental findings, or with haemoptysis, cough, chest pain, or dyspnoea 10. Radiographically, they are often large, homogenous masses with heterogeneous enhancement. In a review of 12 cases, many of the patients presented with large tumours with involvement of the pleura and often with contralateral mediastinal shift 11. Occasionally, PPSS is cystic and presents with a “benign” pneumothorax 8.

Primary pulmonary synovial sarcomas are exceedingly rare to present with endobronchial extension. Seven such reported cases are summarized in Table 1. These cases predominately involved female patients, who presented with symptoms of cough and haemoptysis 9, 11, 12, 13, 14, 15, 16. All cases were treated with surgical resection, and most needed no additional treatment with chemotherapy or radiation 11, 12, 14, 15, 16. These cases had similar outcomes to other cases of synovial sarcoma without endobronchial involvement, with two cases having disease recurrence years after their resection.

**Table 1:** Primary pulmonary synovial sarcoma cases with endobronchial involvement.

Study	Age	Presentation	Clinical features	Treatment	Outcome
Niwa et al. 9	42 y/o female	Haemoptysis	2.5 cm left main bronchus polyposis tumour	Lobectomy and adjuvant chemotherapy	Recurrence after 3 years
Essary et al. 11	69 y/o male	Dyspnoea, cough, haemoptysis × 5 months	3.5 cm, RUL, extending into bronchus	Lobectomy	Had recurrence at 2 years, deceased at 2 years of unrelated cause
Watanabe et al. 12	34 y/o male	Cough and haemoptysis	LLL tumour with endobronchial polypoid mass	Lobectomy	Disease free
Kumar et al. 13	35 y/o female	Cough and dyspnoea	6 cm LUL tumour with intrabronchial extension	Pneumonectomy and Adriamycin‐based chemotherapy	Disease free 1 year after surgery
Jing Jing et al. 14	36 y/o female	Cough and haemoptysis	Endobronchial tumour in left main stem causing complete LLL collapse	Pneumonectomy	Not reported
Tandon et al. 15	42 y/o female	Cough and haemoptysis × 6 months	2.5 × 2 cm RLL mass with endobronchial extension	Lobectomy	Lost to follow up
Al‐Ani et al. 16	68 y/o female	Cough × 3 months	5.6 × 5.4 × 3 cm soft tissue RUL mass with endobronchial component	Lobectomy	Disease free, being followed by serial scans

Synovial sarcomas are of four histological subtypes: biphasic, monophasic spindle, monophasic epithelial, and poorly differentiated (round cell) 8. Monophasic spindle is more common in adults, such as our case. Monophasic spindle synovial sarcoma is composed of relatively uniform spindle cells with elongated nuclei, slightly basophilic cytoplasm, and indistinct cell borders 7. Pathologically, it stains with many markers for sarcomas; it is often positive for vimentin, BCL‐2, keratin, and CD99. Up to 40% stain positive for S‐100 6. Differential diagnosis of monophasic synovial sarcoma includes malignant peripheral nerve sheath tumour, carcinosarcoma, sarcomatoid carcinoma, and solitary fibrous tumour. The biphasic type contains both epithelial and spindle cells in varying proportions. The differential diagnosis of biphasic synovial sarcoma includes fibrosarcoma and malignant mesothelioma 8. The most important criterion for confirmation of diagnosis of either subtype is the presence of the t(X;18)(p11.2;q11.2) translocation detected by PCR or fluorescence in‐situ hybridization with 96% sensitivity and 100% specificity 7. The translocation results most commonly in two fusion genes: SS18‐SSX1 and SS18‐SSX2; however, many others have been described 8. SS18‐SSX1 is almost always associated with biphasic synovial sarcoma, unlike in our case.

Poor prognostic features of synovial sarcoma include size >5 cm, male gender, age >20 years, extensive tumour necrosis, large number of mitotic figures, neurovascular invasion, and SS18‐SSX1 variant 6. High‐grade, aggressive sarcomas are associated with 5‐ and 10‐year survival rates of 60 and 50%, respectively. Resected tumours often have late local recurrence and metastasis.

Given the rarity of the disease, there are no treatment guidelines. However, patients are typically treated with multimodal therapy involving surgery, radiotherapy, and chemotherapy 6. Wide surgical excision is the treatment of choice in adults with localized disease and is often combined with chemotherapy and radiation in more advanced disease. Synovial sarcomas are more sensitive to chemotherapy compared to other sarcomas, particularly to alkylating agents, such as ifosofamide and doxorubicin. Vascular endothelial growth factor (VEGF) inhibitors such as pazopanib have also been approved for use in synovial sarcoma 8. Majority of pulmonary synovial sarcomas reported in the literature have been treated with surgical resection, followed by doxorubicin‐based chemotherapy. Due to the tendency of late recurrence and metastasis, long‐term follow up for 10 years or more is mandatory 8.

### Disclosure Statement

Appropriate written informed consent was obtained for publication of this case report and accompanying images.
